# The role and implications of RNAscope and mRNA in the diagnosis of tuberculosis

**DOI:** 10.1016/j.ebiom.2024.105230

**Published:** 2024-07-02

**Authors:** Ritah F. Nakiboneka, Wilber Sabiiti

**Affiliations:** a Division of Infection and Global Health, School of Medicine, University of St Andrews, UK; b Department of Pathology, Kamuzu University of Health Sciences, Blantyre, Malawi; c Helse Nord Tuberculosis Initiative (HNTI), Pathology Department, Kamuzu University of Health Sciences, Blantyre, Malawi; d Uganda Virus Research Institute, Entebbe, Uganda

The biology and pathogenesis of tuberculosis (TB) remains an enigma despite being one of the oldest human diseases. The aetiological agent, *Mycobacterium tuberculosis* (Mtb) is a master of living with the host in most cases for life without causing disease. Consequently, a quarter of the world population are estimated to be latently infected with Mtb.^1^ The mechanisms by which Mtb stays latent or progresses to disease in some people and not others are yet to be fully understood. Currently, diagnosis mainly depends on identification of the pathogen structural or chemical characteristics through microscopy, culture, or molecular tests. This diagnostic approach has been quite successful with pulmonary TB diagnosis but less useful with other forms of TB that require non-sputum samples.^2^, ^3^, ^4^ Apart from urine and stool, obtaining good quality extrapulmonary specimens is quite invasive, and expensive to perform. Furthermore, while molecular diagnostics have demonstrated higher sensitivity and specificity, DNA-based molecular tests such as Xpert MTB/RIF are reported to detect dead bacilli because DNA molecule is stable and survives long after cell death.^5^ This leaves RNA-based tests as the closest to detection of live Mtb bacilli.

In a recent *eBiomedicine* issue, Nargan et al.^6^ multiplexed RNAscope and Ziehl-Neelsen (ZN) staining/immunohistochemistry to detect Mtb mRNA in ante- and post-mortem human tissues and Acid-fast bacilli (AFB)- vis-à-vis non-AFB- producing antigen 85B (Ag85B). Intact- and disintegrated- Mtb bacilli coupled with intra- and extra-cellular Mtb mRNA were found. Presence of free Mtb mRNA in host cytoplasm and extra cellular spaces raises the question on how long it takes for RNA to be degraded by RNAses or other mechanisms within the host. The conventional thinking is that RNA gets degraded soon after being released from cells. And our work using Mtb 16S rRNA as a surrogate measure for sputum bacillary load showed that rRNA reduces with treatment implying cells being killed and RNA degraded.^7^ However, Nargan et al.'s study has shown that a measure of TB biomarkers in sputum may not reflect clearance of bacilli in tissues and that mRNA could persist long after cell death. The detection of Mtb mRNA in tissues following 6–9 months of treatment further confirms that the tools used to diagnose the patients were ineffective as pertains to detection of Mtb residing in tissues. This opens opportunity for further research into the degradation mechanism of free pathogen mRNA in the host and whether detection of this free RNA in tissues has any pathological and/or clinical significance.

From a basic science perspective, mRNA is required for normal cell function and is a sign of metabolically active cells. Thus, its identification and specificity would translate into adequate identification of the pathogen. However, it has been reported that ZN negative Mtb phenotype *in-vivo* may be the dormant phenotype of Mtb bacilli associated with latent TB infection.^8^ Thus, existence of mRNA in tissues may reflect a low-level metabolism occurring in dormant bacilli. Studies have further shown that Mtb has ability to alter its transcription rate in harsh environments through delaying the decay rate of its mRNA pool, and that hypoxia resulted into a more global stabilization of transcripts.^9^ RNAscope combined with immunohistochemistry provided an extra level of resolution to detect Mtb bacilli in an environment where the standard-of-care tools are unable to reach. The downside is that the method is quite invasive and labour intensive and may be unimplementable in most routine healthcare facilities in low resource settings. Furthermore, evaluation of specificity of the test using a sterile neonatal lung tissue is good for analytical specificity but not real-world specificity which requires an adult lung colonised by other bacteria including non-tuberculous mycobacteria. This would prove the method's ability to detect Mtb mRNA, distinguishing it from other non-Mtb mRNA. Future longitudinal studies assessing how the biomarker expression in human tissues compares to other measures of cell viability will further elucidate its specificity and clinical significance.

## Contributors

R.N. and W.S. performed the literature search, interpretated the evidence and wrote the commentary. Both authors read and approved the final manuscript.

## Declaration of interests

The authors declare no competing interests.

## References

[1] Kiazyk S., Ball T.B. (2017). Latent tuberculosis infection: an overview. Can Commun Dis Rep.

[2] Donovan J., Thu D.D.A., Phu N.H. (2020). Xpert MTB/RIF ultra versus Xpert MTB/RIF for the diagnosis of tuberculous meningitis: a prospective, randomised, diagnostic accuracy study. Lancet Infect Dis.

[3] Kumar P., Sen M.K., Chauhan D.S., Katoch V.M., Singh S., Prasad H.K. (2010). Assessment of the N-PCR assay in diagnosis of pleural tuberculosis: detection of M.tuberculosis in pleural fluid and sputum collected in Tandem. PLoS One.

[4] Mohammed Elbrolosy A., El Helbawy R.H., Mansour O.M., Latif R.A. (2021). Diagnostic utility of GeneXpert MTB/RIF assay versus conventional methods for diagnosis of pulmonary and extra-pulmonary tuberculosis. BMC Microbiol.

[5] Arend S.M., van Soolingen D. (2018). Performance of Xpert MTB/RIF Ultra: a matter of dead or alive. Lancet Infect Dis.

[6] Nargan K., Glasgow J.N., Nadeem S. (2024). Spatial distribution of Mycobacterium tuberculosis mRNA and secreted antigens in acid-fast negative human antemortem and resected tissue. eBioMedicine.

[7] Musisi E., Wamutu S., Ssengooba W. (2024). Accuracy of the tuberculosis molecular bacterial load assay to diagnose and monitor response to anti-tuberculosis therapy: a longitudinal comparative study with standard-of-care smear microscopy, Xpert MTB/RIF Ultra, and culture in Uganda. Lancet Microbe.

[8] Vilchèze C., Kremer L. (2017). Acid-fast positive and acid-fast negative Mycobacterium tuberculosis: the koch paradox. Microbiol Spectr.

[9] Rustad T.R., Minch K.J., Brabant W. (2013). Global analysis of mRNA stability in Mycobacterium tuberculosis. Nucleic Acids Res.

